# Integrated nomogram based on five stage-related genes and TNM stage to predict 1-year recurrence in hepatocellular carcinoma

**DOI:** 10.1186/s12935-020-01216-9

**Published:** 2020-04-29

**Authors:** Haohan Liu, Yongcong Yan, Ruibing Chen, Mengdi Zhu, Jianhong Lin, Chuanchao He, Bingchao Shi, Kai Wen, Kai Mao, Zhiyu Xiao

**Affiliations:** 1 Guangdong Provincial Key Laboratory of Malignant Tumor Epigenetics and Gene Regulation, Sun Yat-Sen Memorial Hospital, Sun Yat-Sen University, Guangzhou, 510120 China; 2 Department of Hepatobiliary Surgery, Sun Yat-Sen Memorial Hospital, Sun Yat-Sen University, Guangzhou, 510120 China

**Keywords:** Hepatocellular carcinoma, 1-year recurrence, Risk score model, Nomogram, TNM stage

## Abstract

**Background:**

The primary tumor, regional lymph nodes and distant metastasis (TNM) stage is an independent risk factor for 1-year hepatocellular carcinoma (HCC) recurrence but has insufficient predictive efficiency. We attempt to develop and validate a nomogram to predict 1-year recurrence in HCC and improve the predictive efficiency of the TNM stage.

**Methods:**

A total of 541 HCC patients were enrolled in the study. The risk score (RS) model was established with the logistic least absolute shrinkage and selector operation algorithm. The predictive nomogram was further validated in the internal testing cohort and external validation cohort. The area under the receiver operating characteristic curves (AUCs), decision curves and clinical impact curves were used to evaluate the predictive accuracy and clinical value of the nomogram.

**Results:**

In the training cohort, we identified a RS model consisting of five stage-related genes (NUP62, EHMT2, RANBP1, MSH6 and FHL2) for recurrence at 1 year. The 1-year disease-free survival of patients was worse in the high-risk group than in the low-risk group (*P *< 0.0001), and 1-year recurrence was more likely in the high-risk group (Hazard ratio: 3.199, *P *< 0.001). The AUC of the nomogram was 0.739, 0.718 and 0.693 in the training, testing and external validation cohort, respectively, and these values were larger than the corresponding AUC of the TNM stage (0.681, 0.688 and 0.616, respectively).

**Conclusions:**

A RS model consisting of five stage-related genes was successfully identified for predicting 1-year HCC recurrence. Then, a novel nomogram based on the RS model and TNM stage to predict 1-year HCC recurrence was also developed and validated.

## Background

Primary liver cancer is predicted to be the sixth leading diagnosed cancer and the fourth leading cause of cancer-related death worldwide. In 2018, an estimated 841,080 new cases of liver cancer (4.7% of all sites) occurred around the world, while approximately 781,631 deaths (8.2%) occurred. Hepatocellular carcinoma (HCC) is the most common pathological type (75–85%) of primary liver cancer [1]. Currently, surgical resection is considered the dominant curative treatment for resectable HCC, compared with other treatments, such as radiofrequency ablation (RFA), transcatheter arterial chemoembolization (TACE) and transplantation [2]. Although the overall survival (OS) of HCC patients could be improved by surgical resection, approximately 60% of patients experience recurrence or distant metastasis after surgery [3, 4]. Postoperative recurrence can be categorized as early or late; the former is much more common and indicates a worse prognosis along with aggressive biological characteristics of the tumor [5]. Early recurrence (< 1 year) after liver resection for HCC is the leading cause of death during the first 2 years [6] and represents an risk factor for poor survival [7]. Therefore, it is essential to determine available predictors of early recurrence so that therapeutic and follow-up management strategies can be adjusted to possibly prevent HCC relapse.

According to the seventh edition cancer staging manual of the American Joint Committee on Cancer (AJCC), the stage of HCC samples ranges from stage I to stage IV with increasing malignancy [8]. The primary tumor, regional lymph nodes and distant metastasis (TNM) stage has been found to have predictive value for early recurrence [9]. However, The Cancer Genome Atlas (TCGA) liver hepatocellular carcinoma (LIHC) cohort shows that the predictive efficiency of the TNM stage, which is an independent risk factor for early HCC recurrence, is still insufficient, resulting in limited utility of the TNM stage as a predictor for early recurrence. Therefore, a risk score (RS) model for 1-year HCC recurrence was developed by the logistic least absolute shrinkage and selector operation (LASSO) algorithm in our study, as the four-gene signature for HCC successfully identified in our previous study [10]. The nomogram is a clear and simple computing model that is used to change the weight of risk factors for the study endpoint into visually linear points [11]. To date, no nomogram combining an RS model and the TNM stage has been applied to predict 1-year HCC recurrence. Therefore, we used HCC samples from the TCGA to establish the RS model and nomogram. In addition, to illustrate the clinical utility of the model, decision curve analysis (DCA) and clinical impact curve analysis were implemented.

Nowadays, the detective methods have become more and more abundant, such as the development of primer kits for some specific genes or non-coding RNAs, and the application of RNA sequencing or gene microarray technology. All of them could have important roles for predicting the prognosis of patients. However, because of the longer time and higher cost of RNA sequencing or microarray, HCC patients would rather to choose a detective method which is faster, more economical and more feasible. Through the screening of global public data, five genes that significant for 1-year recurrence of HCC recurrence were found. By establishing a RS model and constructing a nomogram, we could predict 1-year recurrence of HCC patients and visualize it in a simple way.

Our study aimed to establish a validated, novel nomogram based on the RS model and TNM stage to predict 1-year recurrence in HCC and improve the predictive efficiency of the TNM stage.

## Methods

### Patients and data collection

The discovery cohort contained 60 HCC samples from Iizuka liver 2, a dataset of the Oncomine database (https://www.oncomine.org). We downloaded the mRNA high-throughput sequencing data and clinical data of 374 HCC samples and 50 normal samples from the TCGA (https://portal.gdc.cancer.gov/). After excluding samples with missing information, such as the disease-free survival (DFS) time, TNM stage and histological grade, the remaining number of samples in the entire TCGA LIHC cohort was 274. The samples were randomly assigned to a training cohort with *N *× *q* samples and a testing cohort with *N *× (1 − *q*) samples (*q *= 2/3). We further searched for an external validation cohort and used 107 HCC samples with recurrence-free survival data from GSE76427 to validate our results. Microarray data and patient clinical information of GSE76427 were downloaded from the Gene Expression Omnibus database (GEO, https://www.ncbi.nlm.nih.gov/geo/).

### Identification of differentially expressed genes (DEGs)

We downloaded the DEGs related to recurrence at 1 year (*P *< 0.01) and advanced stages (*P *< 0.01) from the Oncomine database, Iizuka liver 2. The DEGs related to recurrence at 1 year were generated from the microarray data of 20 patients with 1-year recurrence versus 40 patients without 1-year recurrence. Similarly, the DEGs related to advanced stages consisted of the microarray data of 10 patients with stage III disease versus 50 patients with stage I/II disease. All upregulated and downregulated genes filtered by combinational analysis of DEGs related to recurrence at 1 year and advanced stages were defined as DEGs related to recurrence at 1 year and advanced stages. In addition, tumor/normal DEGs were identified as those showing a fold change (FC) ≥ 1.5 and *P* value < 0.05 among the 374 tumor tissues and 50 normal tissues in the TCGA LIHC cohort. We identified tumor/normal DEGs using gene expression profiling interactive analysis (GEPIA, http://gepia.cancer-pku.cn/). Finally, the DEGs screened by 1-year DFS analysis (*P *< 0.05) were identified as stage-related genes. All DEGs were listed in Additional file 1: Table S1.

### Survival analysis and gene set enrichment analysis (GSEA)

One-year DFS analysis (Kaplan–Meier analysis) in the training cohort and determination of the best cutoff value of the RS were both performed in X-tile (version 3.6.1). SangerBox (version 1.0.9) was used for the GSEA of different Kyoto Encyclopedia of Genes and Genomes (KEGG) pathways (*P *< 0.05) between the high-risk group and the low-risk group.

### Development of the RS model

To illustrate significant values for the 16 stage-related genes, we used the LASSO algorithm to filter candidate genes that were reliably associated with 1-year HCC recurrence in the training cohort. LASSO penalized regression was conducted in R studio (version 3.6.1) with the glmnet package. The RS was calculated using the sum of the screened stage-related gene expression values weighted by the coefficients (*β*) from the LASSO regression model. We calculated the prognostic RS for each patient according to the following formula [12, 13]:

$${\text{RS}} = {\text{Expression }}\left( {{\text{gene}}1} \right) \, \times \beta \left( {{\text{gene}}1} \right){ + } \cdots {\text{ + expression }}\left( {{\text{gene}}N} \right) \times \beta \left( {{\text{gene}}N} \right).$$

### Construction and validation of nomogram

We used univariate and multivariate Cox analyses to identify independent risk factors for predicting 1-year HCC recurrence in the training cohort. The nomogram was constructed in R studio (Version 3.6.1) with the rms, foreign, nricens and ROCR packages [14, 15]. The term “Points” represents the score of each variable under different values, while “Total Points” means the total score of the collection after the sum of the corresponding individual fractions of all variables. And the term “Linear Predictor” is a coordinate axis of the linear predicted value. The linear predicted value is converted to the corresponding probability value by a certain transformation function. The testing cohort and external validation cohort were used to evaluate the predictive reliability and accuracy of the nomogram. The predictive performance of the nomogram was measured by receiver operating characteristic (ROC) curve analysis, DCA and clinical impact curve analysis. ROC curve analysis was performed by MedCalc (version 15.6.1), while DCA and clinical impact curve analysis were also accomplished in R studio with the rmda package. The clinical utility of the nomogram was determined by DCA after calculating the net benefits at each risk threshold probability [16, 17]. Additionally, we plotted the clinical impact curve of the nomogram in all three cohorts to demonstrate the significance of the nomogram [18].

### Statistical analysis

Univariate and multivariate Cox regression analyses were performed in IBM SPSS Statistics (version 24), which was also used to generate hazard ratios (HRs) and 95% confidence intervals (CIs). All variables were screened using the forward stepwise selection method in a multivariate binary logistic regression model [19]. Kaplan–Meier survival curves were used to estimate 1-year DFS, and survival differences were assessed by a two-sided log-rank test in GraphPad Prism (version 6.0). All statistical tests were two-sided, and a *P*-value < 0.05 was considered statistically significant.

## Results

### Independent risk factors for 1-year recurrence

First, we calculated the rate of 1-year recurrence, 2-year recurrence and overall recurrence in the TCGA HCC samples with DFS data (n = 274). A total of 33.9% (93/274) of the samples showed HCC recurrence at 1 year, 45.6% (125/274) showed HCC recurrence at 2 years, and the overall rate of HCC recurrence was 54.4% (149/274). Among 149 samples that showed recurrence, more samples showed recurrence within 1 year (62.4%, 93/149) than between 1 and 2 years (21.4%, 32/149). In these 149 samples, the time to recurrence ranged from 0.12 to 90.08 months, and the median time to recurrence was 8.75 months (Fig. 2a). Therefore, we considered that it was more suitable and accurate to define early recurrence by recurrence at 1 year in the TCGA cohort.

**Fig. 1:**
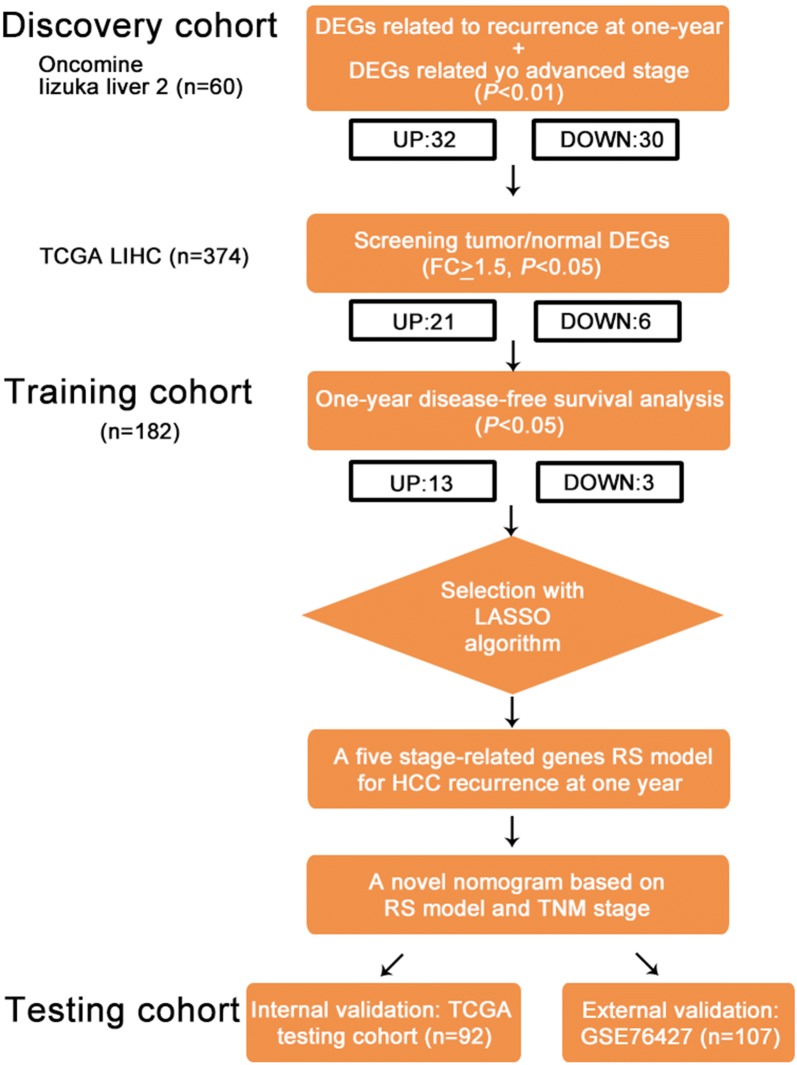
Study flowchart. The design and procedure of our study were shown. Stage-related genes are screened from discovery cohort (n = 434). Novel RS model and nomogram are constructed with TCGA training cohort (n = 182). Testing cohort consists of internal validation cohort (n = 92) and external validation cohort (n = 107)

**Fig. 2:**
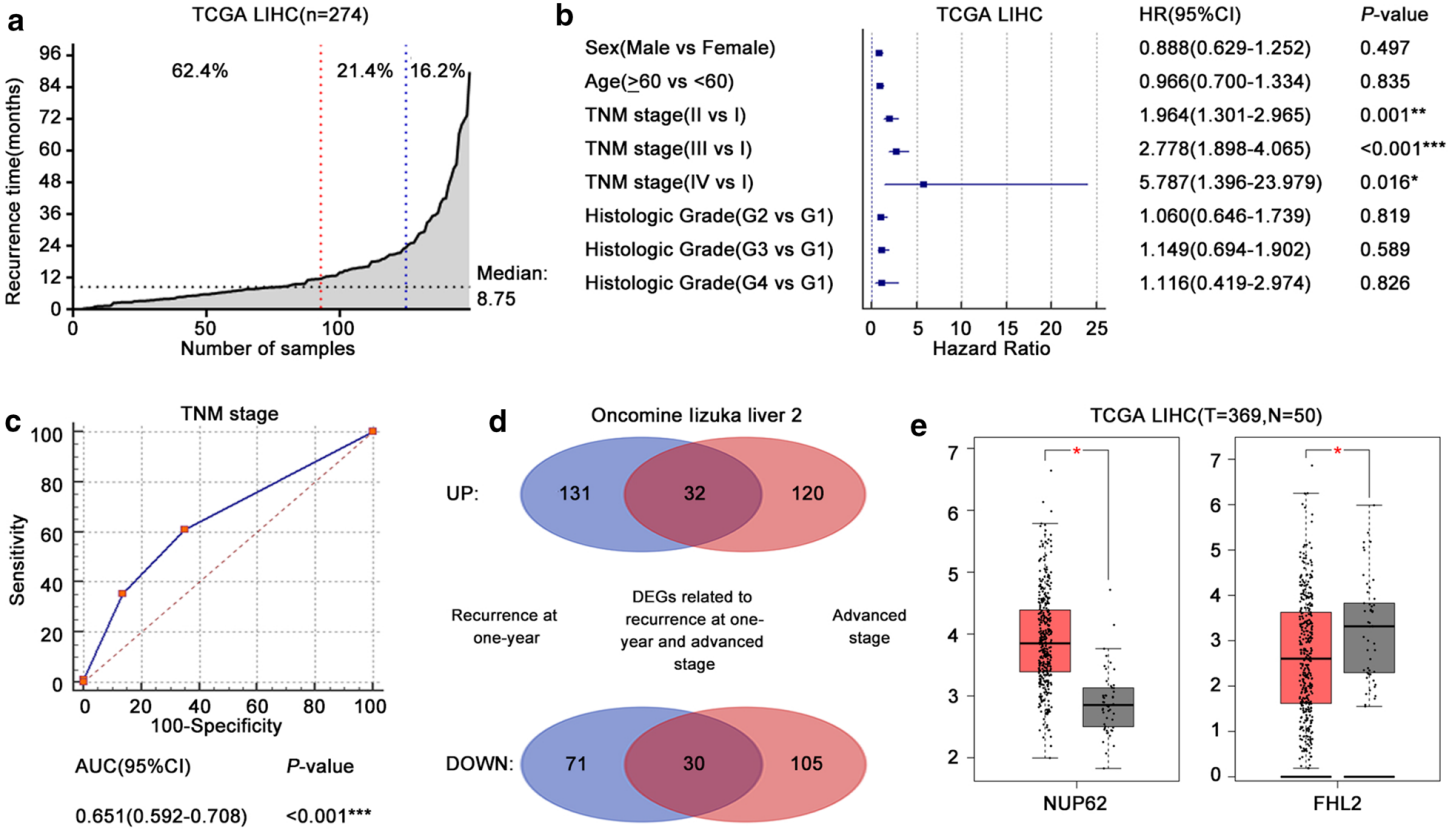
Independent risk factors for 1-year recurrence and stage-related gene identification in the discovery cohort. **a** The red line vertical to the X axis highlights the 1-year cutoff for samples showing recurrence, and the blue line highlights the 2-year cutoff. The black line vertical to the Y axis indicates the median recurrence time. **b** HRs and 95% CIs for risk factors are respectively represented by blue blocks and lines. **c** The ROC curve of the TNM stage for predicting 1-year recurrence was plotted, and the AUC was calculated. **d** Blue circles represent DEGs associated with recurrence at 1 year, and red circles represent DEGs associated with advanced stages. Purple areas indicate overlapping DEGs, and the counts of DEGs are shown. **e** Representative box plots of tumor/normal DEGs are shown. Red boxes: tumors, gray boxes: normal tissues, black dots: samples, black bars: means and standard deviations. *P < 0.05, **P < 0.01, ***P < 0.001

To identify risk factors for 1-year recurrence in the TCGA LIHC samples, we evaluated clinical characteristics by univariate Cox analysis (Additional file 1: Table S2) and plotted them, as shown in Fig. 2b. We found that the TNM stage was the only risk factor for 1-year recurrence with a significant difference. Compared with stage I, the HR (95% CI) of stage II, stage III and stage IV was 1.964 (1.301–2.965), 2.778 (1.898–4.065) and 5.787 (1.396–23.979), respectively. All of the *P*-values were less than 0.05. However, the area under the ROC curves (AUC) of the TNM stage in predicting 1-year recurrence was 0.651, which was still considered to represent low predictive efficacy (Fig. 2c). Therefore, different analyses of DEGs, as shown in the flowchart (Fig. 1), were performed in our study with the aim of enhancing the predictive efficacy of the TNM stage.

### Stage-related gene identification

First, we downloaded DEGs related to recurrence at 1 year (*P *< 0.01) and advanced stages (*P *< 0.01) from the Oncomine database, Iizuka liver 2. After combinational analyses of the upregulated and downregulated DEGs, there were 32 upregulated and 30 downregulated genes related to recurrence at 1 year and advanced stages (Fig. 2d) in the discovery cohort. Next, we used GEPIA to analyze DEGs between tumors and normal tissues among the 374 tumor tissues and 50 normal tissues in the TCGA LIHC cohort (Fig. 2e). Twenty-seven tumor/normal DEGs remained after screening by FC ≥ 1.5 and *P*-value < 0.05. For predicting 1-year recurrence more precisely, every single DEG involved in subsequent analysis should be correlated with 1-year DFS in the TCGA training cohort (n = 182). The clinical characteristics of the training and testing cohorts are shown in Table 1, and there were no significant differences in characteristics between the two cohorts. The high expression of upregulated DEGs was considered to be positively correlated with poor 1-year DFS, as was the low expression of downregulated DEGs. Finally, 16 stage-related genes, including 13 upregulated genes and three downregulated genes, were identified by significant differences (*P *< 0.05) in the 1-year DFS analysis. All DEG lists are shown in Additional file 1: Table S1.

**Table 1:** Clinical characteristics of the training cohort and testing cohort

Clinical characteristics	Entire TCGA cohort (n = 274)	Chi square test		
		Training cohort (n = 182)	Testing cohort (n = 92)	*P*-value
Sex				
Female	83	56	27	0.809
Male	191	126	65	
Age				
< 60	134	94	50	0.395
≥ 60	140	98	42	
TNM stage				
I	149	93	46	0.303
II	65	38	27	
III	68	50	18	
IV	2	1	1	
Histologic grade				
G1	37	25	12	0.685
G2	130	88	42	
G3	97	61	36	
G4	10	8	2	

*TCGA* The Cancer Genome Atlas, *TNM* primary tumor, regional lymph nodes and distant metastasis

### RS model building in the training cohort

**Fig. 3:**
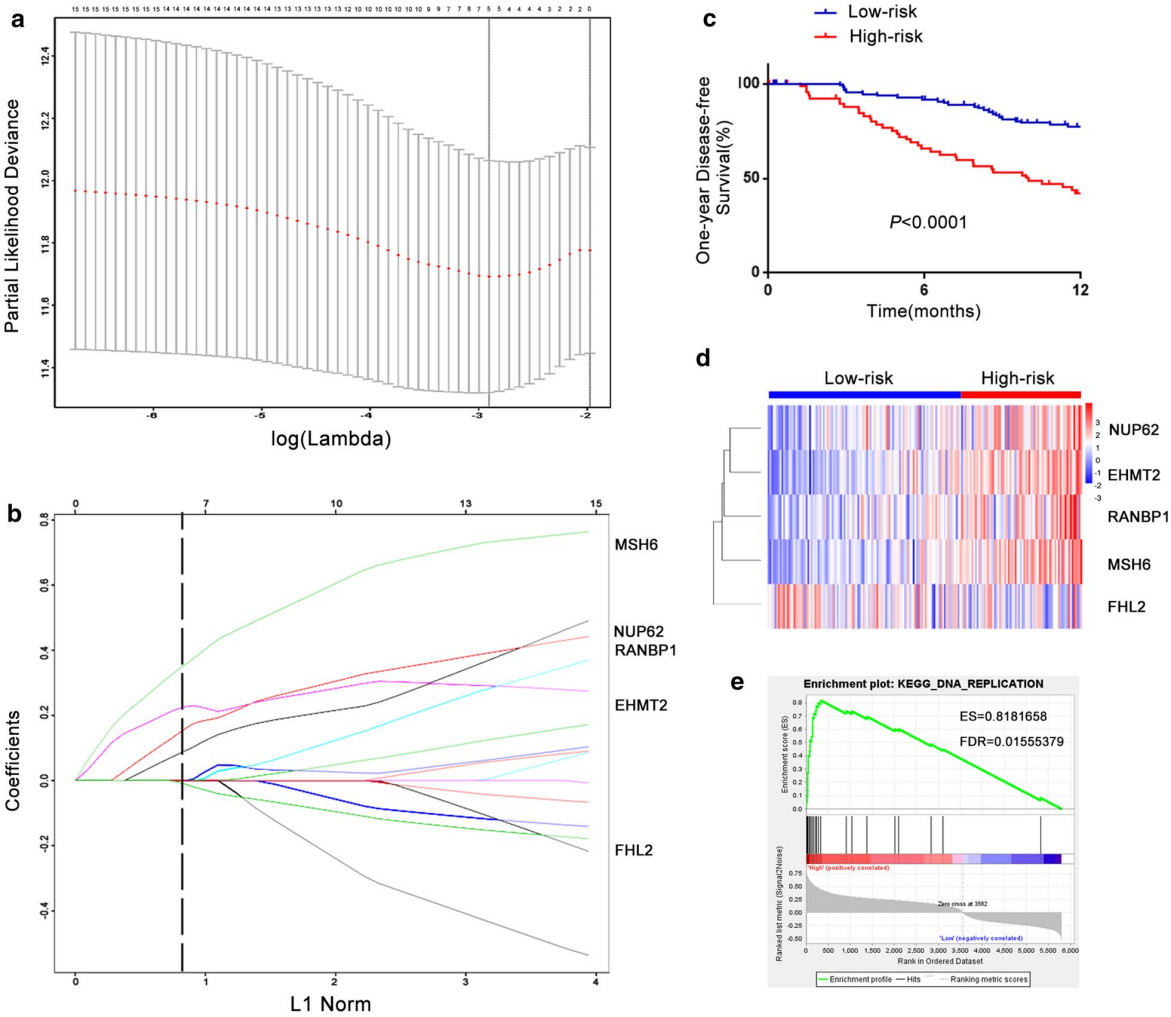
Construction of the prognostic RS model in the training cohort. **a** Selection of the tuning parameter (lambda) in the LASSO model by tenfold cross-validation based on minimum criteria for 1-year recurrence; the lower X axis shows the log (lambda), and the upper X axis shows the average number of stage-related genes. The Y axis indicates the partial likelihood deviance error. The red dots represent the average partial likelihood deviances for every model with a given lambda, and the vertical bars indicate the upper and lower values of the partial likelihood deviance error. The vertical gray lines define the optimal values of lambda, which provide the best fit. **b** LASSO coefficient profiles of 16 stage-related genes. The vertical black dotted lines are plotted at the value selected. **c** Kaplan–Meier analysis of 1-year DFS between the high-risk group (red) and the low-risk group (blue). **d** Heat map of five stage-related genes in the prognostic signature. **e** Representative GSEA plot (DNA replication KEGG pathway) of the high-risk group versus the low-risk group. ES and FDR were also shown

Sixteen stage-related genes were identified after the combinational analysis, differential expression analysis of tumor/normal samples and 1-year DFS analysis. Then, the LASSO regression model was used to further identify candidate genes associated with 1-year HCC recurrence in the TCGA training cohort. As a result, 5 genes were identified: NUP62, EHMT2, RANBP1, MSH6 and FHL2 (Fig. 3a, b). Among them, NUP62, EHMT2, RANBP1 and MSH6 were upregulated stage-related genes, while FHL2 was a downregulated stage-related gene. We further established an RS model with each gene coefficient weighted by LASSO regression. The RS was calculated for each patient in the training cohort as follows:

$$\begin{aligned} {\text{RS}} = \, 0.0 9 2 8 9 3 3 8*\left( {\text{expression level of NUP62}} \right) \, + \, 0. 2 2 80 3 2 8 4*\left( {\text{expression level of EHMT2}} \right) \, + \, 0. 1 60 5 4 8 3 5*\left( {\text{expression level of RANBP1}} \right) \hfill \\ \, + \, 0. 3 5 7 7 1 5 8 4*\left( {\text{expression level of MSH6}} \right) \, - \, 0.0 1 30 1 1 2 2*\left( {\text{expression level of FHL2}} \right). \hfill \\ \end{aligned}$$

After evaluating the RS for each patient in the training cohort, we used X-tile to determine the best cutoff value for the RS (9.251). A total of 112 HCC samples were assigned to the low-risk group, while the high-risk group contained 70 samples. Patients in the high-risk group exhibited worse 1-year DFS than those in the low-risk group, as shown in Fig. 3c (*P *< 0.0001). In addition, the expression levels of NUP62, EHMT2, RANBP1 and MSH6 were higher in the high-risk group than in the low-risk group, while FHL2 exhibited lower expression in the high-risk group (Fig. 3d). Moreover, the DEGs (*P *< 0.05) between the high-risk group and the low-risk group were screened for further GSEA of different KEGG pathways. There were five GSEA reports with a significant difference [False discovery rate (FDR) < 0.05], including DNA replication, cell cycle, mismatch repair, oocyte meiosis and homologous recombination (Additional file 2: Figure S1a–d). Among them, the DNA replication KEGG pathway possessed the highest enrichment score (ES) (Fig. 3e).

### Development of nomogram based on TNM stage and RS

**Fig. 4:**
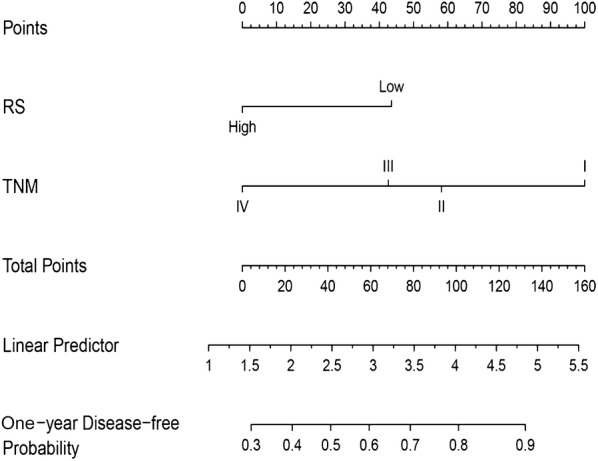
Nomogram based on the TNM stage and RS model for predicting 1-year recurrence in the training cohort. All points assigned on the top point scale for each factor are summed together to generate a total point score. The total point score is projected on the bottom scale to determine the 1-year DFS probability for an individual

Although the RS could successfully distinguish good or poor 1-year DFS between the high-risk group and the low-risk group, the most important purpose of our study was to enhance the predictive efficacy for 1-year recurrence of the TNM stage. It was necessary to perform univariate and multivariate Cox analyses of 1-year recurrence in the training cohort (Table 2). We found that sex (male vs female), TNM stage (stage II vs stage I, stage III vs stage I and stage IV vs stage I) and RS (high vs low) were independent risk factors for 1-year recurrence. However, sex (HR: 0.670, 95% CI 0.397–1.129), stage II (1.949, 0.962–3.949) and stage IV (6.424, 0.816–50.602) were censored in the multivariate Cox analysis (*P *= 0.132, 0.064 and 0.077). The RS exhibited a similar contribution (HR: 3.199, 95% CI 1.891–5.411) as stage III (2.911, 1.616–5.243). Therefore, we developed a nomogram to predict 1-year HCC recurrence based on the TNM stage and RS, as identified in the multivariate Cox analysis (Fig. 4). According to the nomogram, more total points indicates a greater probability of 1-year DFS. Point assignments and predictive scores for each factor in the nomogram were calculated, as shown in Additional file 1: Table S3. The total points range from zero to 143.6 and the 1-year disease-free probability ranges from 0.3 to 0.9. On the basis of high RS and stage IV both corresponding to zero point, the HCC patient with high-risk of RS and stage IV of TNM stage would gain a total point of “0” which represented the 1-year disease-free probability were as worst as 0.3. On the contrary, the low RS and stage I mean higher point, so that the HCC patient with low-risk of RS and stage I of TNM stage would possess a 1-year disease-free probability which more than 0.9.

**Table 2:** Univariate and multivariate Cox analysis of the training cohort

Recurrence at 1 year	Univariate analysis			Multivariate analysis		
	HR	95% CI	*P*-value	HR	95% CI	*P*-value
Sex						
Female						
Male	0.596	0.356–0.996	0.048*	0.670	0.397–1.129	0.132
Age						
< 60						
≥ 60	0.842	0.510–1.391	0.502			
TNM stage						
I						
II	2.011	0.993–4.074	0.052	1.949	0.962–3.949	0.064
III	3.423	1.909–6.139	< 0.001***	2.911	1.616–5.243	< 0.001***
IV	5.163	0.688–38.733	0.110	6.424	0.816–50.602	0.077
Histologic grade						
G1						
G2	0.983	0.447–2.165	0.967			
G3	1.230	0.550–2.751	0.613			
G4	1.259	0.334–4.748	0.734			
RS						
Low						
High	3.416	2.041–5.717	< 0.001***	3.199	1.891–5.411	< 0.001***

*HR* Hazard ratio, *CI* confidence interval, *TNM* primary tumor, regional lymph nodes and distant metastasis, *RS* risk score

*P < 0.05, ***P < 0.001

### Nomogram validation and clinical utility

**Fig. 5:**
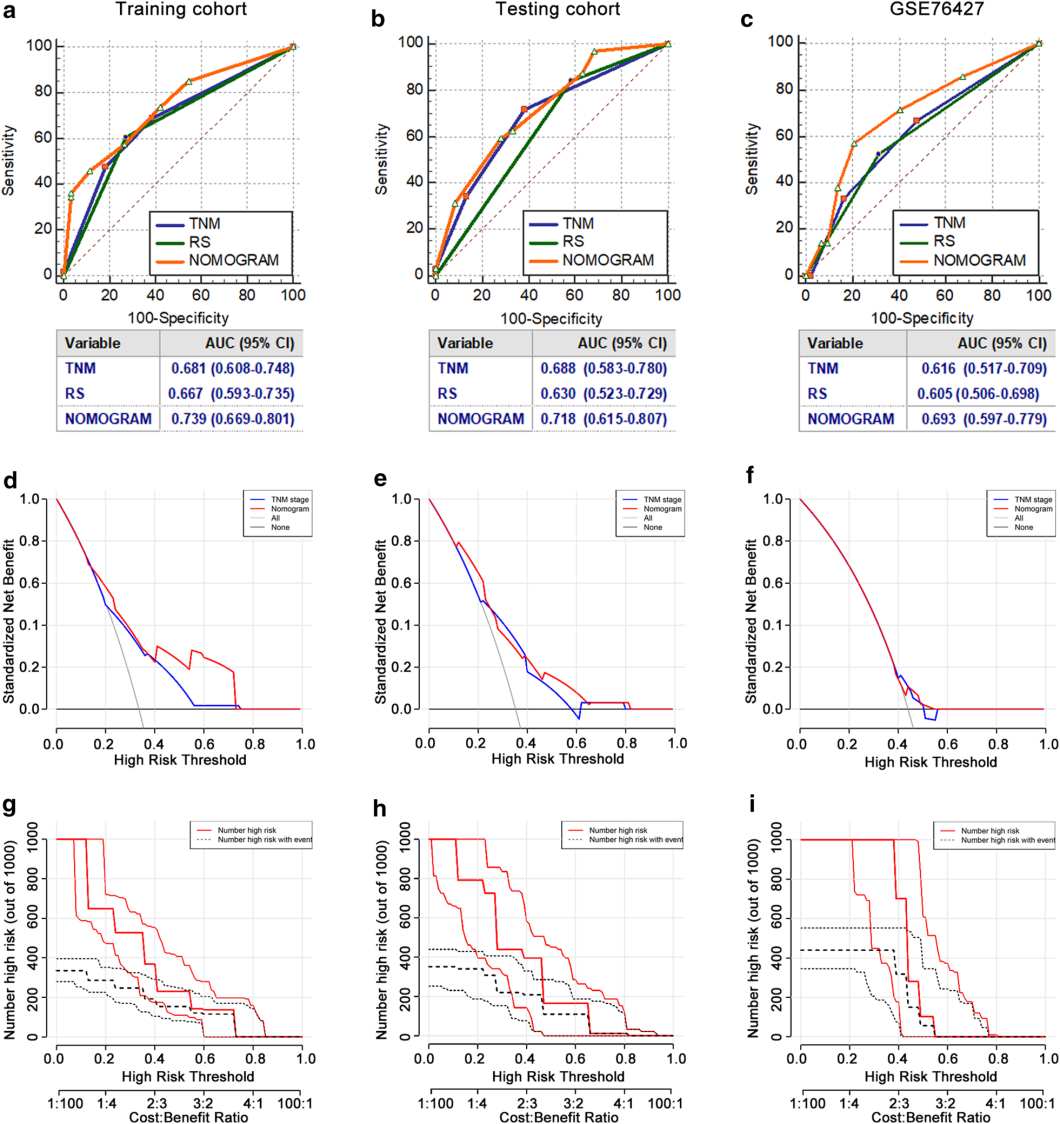
ROC curve analysis, DCA and clinical impact curve analysis in the training cohort, the testing cohort and the external validation cohort. **a–c** Comparisons of the predictive value of the nomogram (orange), TNM stage (blue) and RS (green) for 1-year recurrence according to ROC analysis. ROC curves in the training cohort (**a**), the testing cohort (**b**) and the external validation cohort (**c**). The AUC and 95% CI were calculated. **d**–**f** DCA of the nomogram (red) and TNM stage (blue) for predicting 1-year recurrence in the training cohort (**d**), the testing cohort (**e**) and the external validation cohort (**f**). The X axis shows the high-risk threshold, and the Y axis represents the standardized net benefit. **g–i** Clinical impact curves of the nomogram for predicting 1-year recurrence in the training cohort (**g**), the testing cohort (**h**) and the external validation cohort (**i**). The number of high-risk patients (black dotted line) and the number of high-risk patients with events (red solid line) are plotted

Validation was also necessary after development of the nomogram. First, to compare the predictive values for 1-year recurrence of the nomogram, i.e., the TNM stage and RS, we applied ROC curve analysis. In the plotted ROC curves of the training cohort (Fig. 5a), the AUC of the nomogram was 0.739 (95% CI 0.669–0.801), larger than the AUC of the TNM stage (0.681, 0.608–0.748) and RS (0.667, 0.593–0.735). DCA was performed for the nomogram and TNM stage (Fig. 5d) in the training cohort. A high-risk threshold of 0–0.7 was the most beneficial for predicting 1-year recurrence with our nomogram, which was better than that of the TNM stage. In addition, based on the nomogram DCA, we further plotted curves to evaluate the clinical impact of the nomogram to help us more intuitively understand their substantial value. Clinical impact curves of the nomogram in the training cohort (Fig. 5g) illustrated that the model had remarkable predictive power: the predicted number of high-risk patients was always greater than the number of high-risk patients with 1-year recurrence when the high-risk threshold was in the range of 0–0.7, and the cost–benefit ratio would be acceptable in the same range. In addition, the RS of each patient in the testing cohort and the external validation cohort was also calculated and divided into two groups by the best cutoff value. The AUC of the nomogram in the testing cohort (0.718, 0.615–0.807) and the external validation cohort (0.693, 0.597–0.779), as well as the training cohort, was larger than that of the TNM stage and RS (Fig. 5b, c). We considered that the predictive efficacy of the nomogram was better than that of the TNM stage and RS, and it was successful in enhancing the predictive efficacy of the TNM stage for 1-year recurrence. Compared with the TNM stage, the nomogram showed more benefits in predicting 1-year recurrence by DCA (Fig. 5e, f). However, the high-risk threshold in the external validation cohort ranged from 0 to 0.57, which was smaller than the high-risk threshold in the testing cohort (0–0.8). Clinical impact curves revealed remarkable predictive power for the nomogram. We could declare that the novel nomogram to predict 1-year recurrence was reliably validated by internal and external validation cohorts and had good clinical utility.

## Discussion

Liver cancer ranked sixth in terms of incidence but third in terms of mortality worldwide in 2018, notably ranking fifth in terms of global cases and second in terms of deaths among males [1]. The high mortality rate of HCC is partly due to the incidence of recurrence, among which early recurrence is more common. Nonetheless, there is no consensus regarding the term “early recurrence”. In some previous studies, a period ranging from 6 months to 2 years after curative hepatectomy has been used as a cutoff to define early and late recurrence [20–22]. A study aiming to clarify the optimal duration of early and late recurrence of HCC claimed that 17 months was the best cutoff value [23], which is in contrast with another paper noting that early recurrence likely occurs earlier than previously thought, at 1 or 2 years after hepatectomy [24]. In this case, we decided to determine which threshold was more suitable and accurate in the TCGA cohort.

In our study, almost two-thirds of the samples (62.4%) showed recurrence at 1 year among 149 samples from the overall TCGA cohort with recurrence. The time to recurrence ranged from 0.12 to 90.08 months, and the median time to recurrence was 8.75 months in the 149 samples showing recurrence. These data strongly support the definition of early recurrence as recurrence by 1 year in the TCGA cohort.

Our univariate Cox analysis showed that the TNM stage was a significant independent risk factor for 1-year HCC recurrence. The predictive ability of the TNM stage for tumor recurrence has already been assessed in several types of cancer, such as eyelid carcinoma [25], thymic epithelial tumors [26] and lung cancer [27], and a model has been established combining the TNM stage and tumor size to predict recurrence among HCC patients [28]. However, the AUC of the TNM stage to predict 1-year recurrence in our study demonstrated low predictive efficacy. As a result, we analyzed DEGs related to 1-year recurrence and advanced stages in the Oncomine database. Sixteen stage-related genes were identified after combinational analysis, differential expression analysis of tumor/normal samples and 1-year DFS analysis.

Using the LASSO regression model, an optimal subset of gene-based signatures associated with 1-year HCC recurrence in the TCGA training cohort was selected, among which NUP62, EHMT2, RANBP1, and MSH6 were upregulated and FHL2 was downregulated. NUP62 can promote cell proliferation in squamous cell carcinoma [29], EHMT2 contributes to tumor aggressiveness [30], RANBP1 affects the mitotic microtubule activity and Taxol sensitivity of cancer cells [31], and MSH6 is associated with a poor prognosis in several types of cancer [32], while FHL2 is a negative regulator of TGF-β1 transcription in the liver and plays a suppressive role in HCC metastasis [33, 34]. Based on these previous studies and the RS model established with each gene coefficient weighted by LASSO regression, we considered that the four upregulated genes are likely tumor promoters and that the remaining downregulated gene is a protective gene in HCC.

There have also been several studies on predicting 1-year recurrence in HCC. Zhen Zhang and his group constructed a radiomic nomogram based on gadoxetic acid-enhanced MRI for predicting early postoperative recurrence in HCC [35]. Ying Zhou et al. [36] built a CT-based radiomic signature to identify potential biomarkers for the preoperative prediction of early recurrence in HCC. Another study concluded that tissue miR-125b-5p expression and intrahepatic metastasis were useful for stratifying patients at risk of early HCC recurrence after curative resection [37]. To date, no nomograms based on an RS and the TNM stage have been established for predicting 1-year HCC recurrence.

Therefore, a nomogram was constructed and validated to enhance the predictive efficacy for 1-year recurrence of the TNM stage by incorporating both the RS and TNM stage. The nomogram demonstrated better diagnostic efficiency, as shown by ROC curves and DCA, than the TNM stage and RS in the training and testing cohorts, as well as the external validation cohort. Clinical impact curves also showed that the nomogram had remarkable predictive power and exhibited good clinical utility. In addition, the high-risk threshold of our nomogram ranged from 0 to 0.7 in the training cohort, 0–0.8 in the testing cohort, and 0–0.57 in the external validation cohort according to the nomogram DCA.

Our nomogram based on the RS of five stage-related genes and the TNM stage, a combination of clinical variables and genetic markers, is a novel, reliable and validated model for predicting 1-year recurrence after hepatectomy in HCC. Since TNM stage is an acknowledged staging system worldwide for HCC, and the patients enrolled in our study contain different races and different nationalities such as Japan, America and Singapore. We considered that the nomogram itself had a popularity to some extent. However, we further believed that the nomogram might be applied and validated in different institutions with a larger sample size, so as to truly demonstrate its general applicability. Fortunately, the TNM stage is the basic diagnostic information that can be acquired conveniently, and the expression levels of genes are also easy to be detected. Our nomogram has the potential to become convenient and feasible in clinical practice. However, since the training and testing cohorts in our study were derived from the same TCGA dataset, this could lead to overfitting of the model. And the insufficient of sample size was considered as a significant factor in our nomogram. Additional validation at our hospital or another institution should be performed to verify the availability and accuracy of our model.

## Conclusions

In conclusion, after different analyses of DEGs in the Oncomine dataset based on expression and survival, we screened 16 stage-related genes related to 1-year recurrence. We further applied the LASSO algorithm to develop an RS model consisting of five stage-related genes, which showed an excellent capacity to distinguish 1-year DFS between the high-risk group and the low-risk group. A nomogram based on the RS model and TNM stage for predicting 1-year recurrence was constructed in the training cohort. Moreover, the AUC of the nomogram in the internal testing cohort and the external validation cohort demonstrates that it performs well and has acceptable accuracy and reliability. Our nomogram was demonstrated to be clinically useful by DCA and clinical impact curve analysis. We believe that our nomogram could help clinicians provide improved individual treatment and make clinical decisions, as well as guide follow-up management strategies, for HCC patients who are predicted to have a high probability of 1-year recurrence.

## Supplementary information

**Additional file 1: Table S1.** DEGs list. **Table S2.** Univariate Cox analysis of recurrence at 1 year in the TCGA LIHC dataset. **Table S3.** Points assignment of nomogram factors.

**Additional file 2: Figure S1**. GSEA plot of the high-risk group versus the low-risk group. **a**–**d** Cell cycle (a), mismatch repair (b), oocyte meiosis (c) and homologous recombination (d) KEGG pathway. ES and FDR were also shown.

## Data Availability

The data downloaded from the Oncomine database was needed a paid personal account. The other data were available in the LIHC dataset from the TCGA database and the GSE76427 dataset from the GEO database.

## Abbreviations

HCC: Hepatocellular carcinoma
RFA: Radiofrequency ablation
TACE: Transcatheter arterial chemoembolization
OS: Overall survival
AJCC: American Joint Committee on Cancer
TNM: Primary tumor, regional lymph nodes and distant metastasis
TCGA: The Cancer Genome Atlas
LIHC: Liver hepatocellular carcinoma
RS: Risk score
LASSO: Least absolute shrinkage and selector operation
DCA: Decision curve analysis
DFS: Disease-free survival
GEO: Gene Expression Omnibus
DEGs: Different expression genes
GEPIA: Gene Expression Profiling Interactive Analysis
GSEA: Gene set enrichment analysis
ROC: Receiver operating characteristic
HRs: Hazard ratios
CIs: Confidence intervals
AUC: Area under the receiver operating characteristic curve
FDR: False discovery rate
ES: Enrichment score
