# Supplementary material for: Integrated nomogram based on five stage-related genes and TNM stage to predict 1-year recurrence in hepatocellular carcinoma
Source: Cancer Cell Int. 2020 Apr 29;20:140. doi: 10.1186/s12935-020-01216-9 (PMC7189530; doi:10.1186/s12935-020-01216-9)
Supplement: Supplementary file 1 — Additional file 1: Table S1. DEGs list. Table S2. Univariate Cox analysis of recurrence at 1 year in the TCGA LIHC dataset. Table S3. Points assignment of nomogram factors. [file 12935_2020_1216_MOESM1_ESM.docx]

**Table S1** DEGs list.

| Iizuka Liver 2: DEGs related to recurrence at one year and advanced stage (*P*<0.01) | PDHB, SULT1E1, NUP62, MAGEA5, QARS, GAPDH, HSF1, NEU1, GATA4, MTOR, CANX, CSNK2B, PRKCG, RDBP(NELFE), MLH1, LRPAP1, EHMT2, SRM, DCTN2, RANBP1, PSMD7, MSH6, AKR1C3, TRAP1, SREBF1, CCT3, TGFBR1, TAF5, CCT4, WNT7A, CDK4, CLCNKB (UP)  IL10RA, MRC1, IGF1, HLA-DQA1, GZMA, BTG1, CELF2, IFNGR1, MEF2C, ANK2, IRF9, SGK1, TRIM22, CD55, IRF8, TCEA2, F2R, LAPTM5, ITGB6, MKNK1, IFI44, CFD, FHL2, NUP98, CD74, HLA-DRB1, PNRC1, RQCD1, NR4A2, IFI44L (DOWN) |
| --- | --- |
| TCGA LIHC: tumor/normal DEGs (FC ≥1.5, *P*<0.05) | NUP62, QARS, GAPDH, HSF1, NEU1, MTOR, CANX, CSNK2B, RDBP(NELFE), MLH1, LRPAP1, EHMT2, SRM, DCTN2, RANBP1, PSMD7, MSH6, AKR1C3, CCT3, CCT4, CDK4 (UP)  MRC1, IGF1, SGK1, IRF8, FHL2, NR4A2 (DOWN) |
| TCGA training cohort: Stage-related genes after one-year disease-free survival analysis (*P*<0.05) | NUP62, GAPDH, NEU1, CSNK2B, MLH1, EHMT2, SRM, RANBP1, MSH6, AKR1C3, CCT3, CCT4, CDK4 (UP)  MRC1, IRF8, FHL2 (DOWN) |

DEGs= Differential expressed genes; TCGA= The Cancer Genome Atlas; LIHC= Liver hepatocellular carcinoma; FC= Fold change.

**Table S2** Univariate Cox analysis of recurrence at one year in the TCGA LIHC dataset.

| Recurrence at one year | Univariate Analysis | | |
| --- | --- | --- | --- |
|  | HR | 95%CI | *P*-value |
| **Sex**  Female  Male | 0.888 | 0.629-1.252 | 0.497 |
| **Age**  <60  ≥60 | 0.966 | 0.700-1.334 | 0.835 |
| **TNM stage**  I  II  III  IV | 1.964  2.778  5.787 | 1.301-2.965  1.898-4.065  1.396-23.979 | 0.001**  <0.001***  0.016* |
| **Histologic Grade**  G1  G2  G3  G4 | 1.060  1.149  1.116 | 0.646-1.739  0.694-1.902  0.419-2.974 | 0.819  0.589  0.826 |

***P*<0.01, ****P*<0.001.

TCGA= The Cancer Genome Atlas; LIHC= Liver hepatocellular carcinoma; TNM= Primary tumor, regional lymph nodes and distant metastasis; HR= Hazard ratio; CI= Confidence interval.

**Table S3** Points assignment of nomogram factors.

| Nomogram factors | Points |
| --- | --- |
| **TNM stage**  I  II  III  IV | 100  58.2  42.6  0 |
| **RS**  Low  High | 43.6  0 |

TNM= Primary tumor, regional lymph nodes and distant metastasis; RS= Risk score.
