# Supplementary figures and images for: Integrated nomogram based on five stage-related genes and TNM stage to predict 1-year recurrence in hepatocellular carcinoma
Source: Cancer Cell Int. 2020 Apr 29;20:140. doi: 10.1186/s12935-020-01216-9 (PMC7189530; doi:10.1186/s12935-020-01216-9)

a

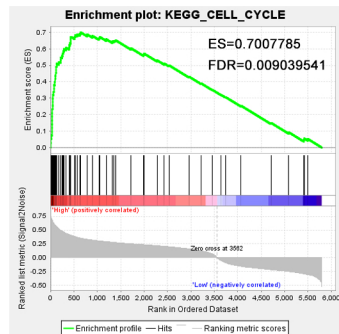

b

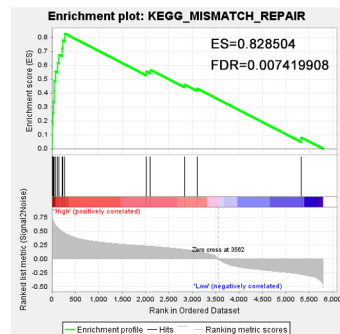

c

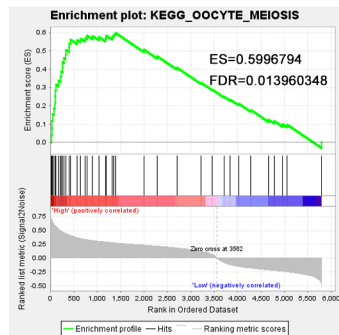

d

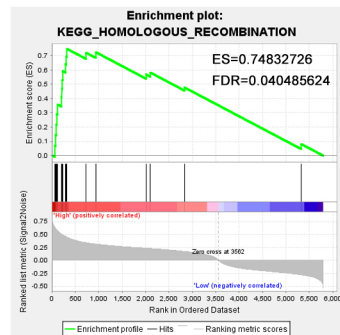

Supplement: Supplementary file 2 — Additional file 2: Figure S1. GSEA plot of the high-risk group versus the low-risk group. a–d Cell cycle (a), mismatch repair (b), oocyte meiosis (c) and homologous recombination (d) KEGG pathway. ES and FDR were also shown. [file 12935_2020_1216_MOESM2_ESM.pdf]
